# Supplementary material for: Ultrafast coherent control of a hole spin qubit in a germanium quantum dot
Source: Nat Commun. 2022 Jan 11;13:206. doi: 10.1038/s41467-021-27880-7 (PMC8752786; doi:10.1038/s41467-021-27880-7)
Supplement: Supplementary file 1 — Supplementary Information [file 41467_2021_27880_MOESM1_ESM.pdf]

Supplementary Information

Ultrafast Coherent Control of a Hole Spin Qubit in a Germanium Quantum Dot

Ke Wang,[1,2,#] Gang Xu,[1,2,#] Fei Gao,[3] He Liu,[1,2] Rong-Long Ma,[1,2] Xin Zhang,[1,2] Zhanning Wang,[4] Gang Cao,[1,2] Ting Wang,[3] Jian-Jun Zhang,[3,*] Dimitrie Culcer,[4] Xuedong Hu,[5] Hong-Wen Jiang,[6] Hai-Ou Li,[1,2,*] Guang-Can Guo,[1,2] and Guo-Ping Guo[1,2,7*]

[1] *CAS Key Laboratory of Quantum Information, University of Science and Technology of China, Hefei, Anhui 230026, China*
[2] *CAS Center for Excellence and Synergetic Innovation Center in Quantum Information and Quantum Physics, University of Science and Technology of China, Hefei, Anhui 230026, China*
[3] *Institute of Physics and CAS Center for Excellence in Topological Quantum Computation, Chinese Academy of Sciences, Beijing 100190, China*
[4] *School of Physics, University of New South Wales, Sydney 2052, Australia*
[5] *Department of Physics, University at Buffalo, SUNY, Buffalo, New York 14260, USA*
[6] *Department of Physics and Astronomy, University of California, Los Angeles, California 90095, USA*
[7] *Origin Quantum Computing Company Limited, Hefei, Anhui 230026, China*

[#] These authors contributed equally to this work.
* Corresponding author. Emails: jjzhang@iphy.ac.cn (J.-J. Z.); haiouli@ustc.edu.cn (H.-O. L.); gpguo@ustc.edu.cn (G.-P.G.).

Supplementary Note 1: Schematic representation of the device and the charge stability diagram
Supplementary Note 2: EDSR spectrum
Supplementary Note 3: Noise power spectral density
Supplementary Note 4: Additional information on the Rabi oscillation of mode A and dephasing time
Supplementary Note 5: Two-axis control of mode B
Supplementary Note 6: Simulation of static electric field E_{dc} and effective driving field E_{ac}
Supplementary Note 7: Calibration of the phase of second pulse in Ramsey fringe pattern
Supplementary Note 8: References on different quantum dot spin systems
Supplementary Note 9: Heavy hole states in GHW
Supplementary Note 10: Heavy hole spins in GHW quantum dots
References

Supplementary Note 1: Schematic representation of the device and the charge stability diagram

Our device consists of a silicon substructure, a nanowire, metal gates and an insulator layer, as illustrated in Supplementary Fig. 1a. Voltages applied to three electrodes create elongated quantum dots along the nanowire (y-direction) under the gates. A static magnetic field is applied perpendicular to the substructure (along the z-direction), where a largest g-factor can be obtained compared to other directions. The charge stability diagram is shown in Supplementary Fig. 1b. Zoom in, Supplementary Fig. 1c shows the squared part in Supplementary Fig. 1b. To confirm the regime of PSB, we measure the "triangle" at reversed bias for comparison (Supplementary Fig. 1d). When the detuning equals zero, a large current (>30 pA) is observed at the bias of -3 mV while it is suppressed (~1 pA) at $V_{\mathrm{sd}} = 3$ mV.

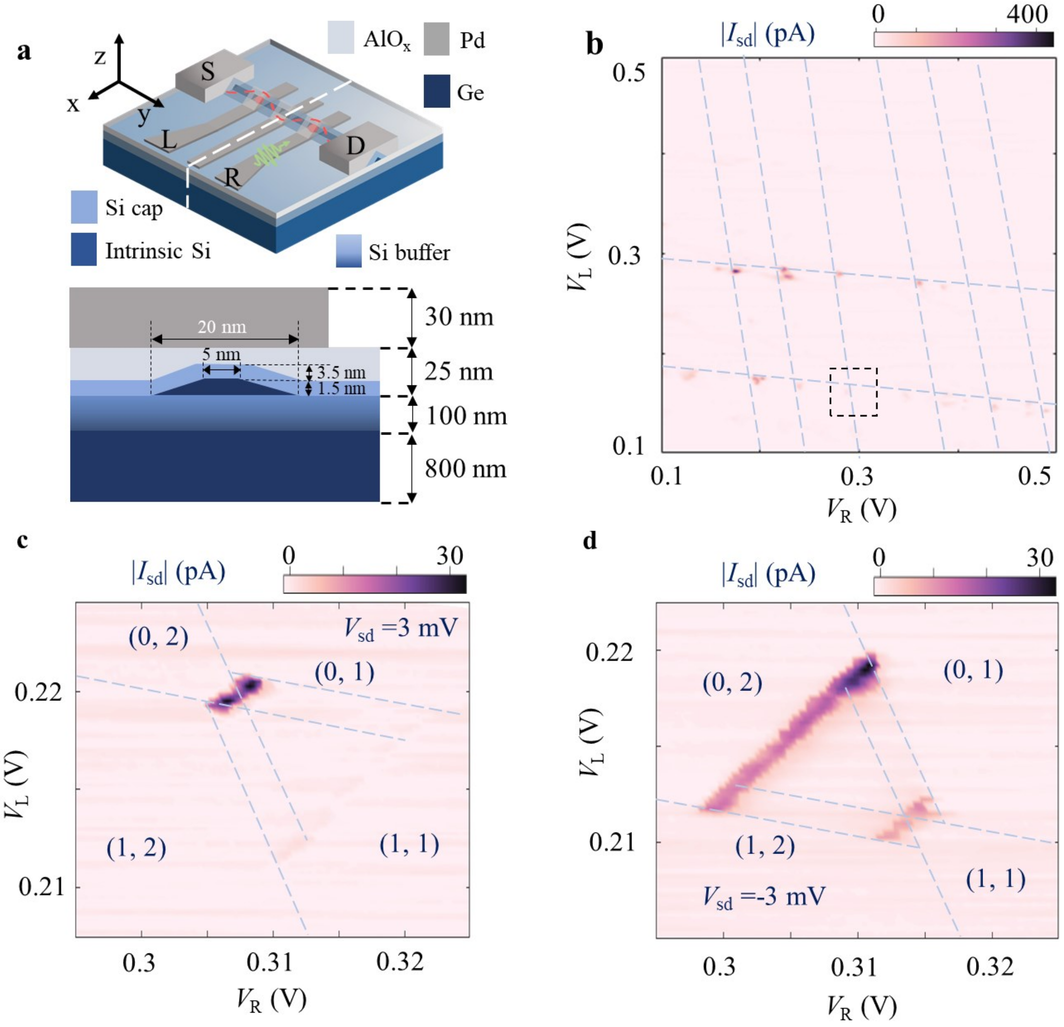

Supplementary Figure 1: Schematic representation of the device and the stability diagram. **a**, Schematic representation of the three-gate device and the cross-section along the dash line. The GHW, which consists of a Si cap and a Ge layer, is grown on Si substrate and covered by a layer of aluminum oxide. Three 35 nm wide gates, spaced at 30 nm, are deposited on top of this insulating layer and HW. **b**. Stability diagram of the double quantum device at $V_{\mathrm{sd}} = 2$ mV. The dashed rectangle is where we measure the conductance triangle. An intra-dot Coulomb energy, i.e. orbital splitting, of 10 meV is extracted from ΔV_{R1}. **c**, The conductance bias triangle of the DQD at $V_{\mathrm{sd}} = 3$ mV (the same as Fig. 1b in the main text) and the inter-dot Coulomb interaction of 0.5 meV obtained from $\Delta \mathrm{V}_{\mathrm{R2}}$. **d**, The conductance bias triangle at the same regime with of a bias of $V_{\mathrm{sd}} = -3$ mV, a large leakage current can be observed at $\varepsilon \sim 0$.

Supplementary Note 2: EDSR spectrum

We measure the EDSR signal by observing the variation in the leakage current in the PSB regime. When EDSR flips a spin, PSB is lifted and the current starts to flow, until another hole with the right spin state blocks the transport again. As such, EDSR leads to an increase in the leakage current. Notice that if none of the hole spins are flipped when driven, PSB cannot be lifted and leakage current would not change. Thus the change in PSB leakage current is a clear indication that a spin-flip transition has happened.

In our measurement, we apply a continuous microwave pulse to one of the gates (R). The electric field E_{ac} that drives EDSR can be estimated from Supplementary Fig. 8c & d by modeling our device and inputting the a.c. voltage V_{ac} from the microwave source. Under different magnetic fields and microwave frequencies, more than 5 oblique lines with anti-crossings are obtained at a low microwave power $P = -15$ dBm (Supplementary Fig. 2a) indicating that the microwave is on resonance with a particular spin-flip transition. In the spectroscopy, drifts in the transport current account for current variation along the longitudinal axis while the step-like signal along the horizontal axis is attributed to the attenuation difference of the circuit.

The most commonly observed Pauli Spin Blockade happens near the $(0,2)-(1,1)$ transition, though it has also been observed in multi-electron double dots[1-3] while our DQD contains roughly five to ten holes in each dot, we find that an effective two-hole model (essentially assuming that core holes do not participate in the low energy dynamics related to PSB) produces a spectrum that fits our observation quite well. In this model, we include basis states of single-dot singlet, S_{02}, and six two-hole states in the (11) regime: $|{\downarrow\uparrow}\rangle$, $|{\uparrow\downarrow}\rangle$, $|{\uparrow\uparrow}\rangle$ (T_+) and $|{\downarrow\downarrow}\rangle$ (T_-) in the ground orbital states, along with two zero-spin states in an excited orbital state $|{\uparrow\downarrow}\rangle_e$ and $|{\downarrow\uparrow}\rangle_e$. Here, only the ground triplets in the (11) regime are considered since T_{02} has much higher energy than S_{02} due to Pauli exclusion principle. The excited triplets are not discussed in our model. We also do not consider $|{\downarrow\downarrow}\rangle_e$ as the transition does not lift PSB and cannot be observed in our experiment. The corresponding two-hole Hamiltonian can be written as

$$
H_{DQD}=\begin{pmatrix}
\Delta_\mathrm{e}-(g_1-g_2)\mu_B B/2 & 0 & -t_\mathrm{e}/\sqrt{2} & 0 & 0 & {\Delta_\mathrm{SO}^\mathrm{e1}}^* & -\Delta_\mathrm{SO}^\mathrm{e1}\\
0 & \Delta_\mathrm{e}+(g_1-g_2)\mu_B B/2 & t_\mathrm{e}/\sqrt{2} & 0 & 0 & {\Delta_\mathrm{SO}^\mathrm{e2}}^* & -\Delta_\mathrm{SO}^\mathrm{e2}\\
-t_\mathrm{e}/\sqrt{2} & t_\mathrm{e}/\sqrt{2} & -\varepsilon & -t/\sqrt{2} & t/\sqrt{2} & \Delta_\mathrm{SO}^* & -\Delta_\mathrm{SO}\\
0 & 0 & -t/\sqrt{2} & -(g_1-g_2)\mu_B B/2 & 0 & 0 & 0\\
0 & 0 & t/\sqrt{2} & 0 & (g_1-g_2)\mu_B B/2 & 0 & 0\\
\Delta_\mathrm{SO}^\mathrm{e1} & \Delta_\mathrm{SO}^\mathrm{e2} & \Delta_\mathrm{SO} & 0 & 0 & (g_1+g_2)\mu_B B/2 & 0\\
-{\Delta_\mathrm{SO}^\mathrm{e1}}^* & -{\Delta_\mathrm{SO}^\mathrm{e2}}^* & -\Delta_\mathrm{SO}^* & 0 & 0 & 0 & -(g_1+g_2)\mu_B B/2
\end{pmatrix}
$$

where g_1 and g_2 are the g-factors of the hole spin in the two dots respectively, t and t_e are the spin-independent tunnel coupling between the two dots, Δ_SO is the spin-flip tunnel coupling between $|\uparrow\uparrow\rangle$ /$|\downarrow\downarrow\rangle$ and S_{02}, $\Delta_\mathrm{SO}^\mathrm{e}$ is the spin-flip tunnel coupling between exctied state and $|\uparrow\uparrow\rangle$ /$|\downarrow\downarrow\rangle$, Δ_e is the energy gap between ground states and excited states, and ε is the detuning of $\mathrm{S}_{11}=1/\sqrt{2}(|\uparrow\downarrow\rangle-|\downarrow\uparrow\rangle)$ with respect to S_{02}.

While in this model we do not assume any particular characteristics for the involved orbital states, the robust spin blockade we have observed is a strong indication that the excited orbital involved in the (1,1) excited state is localized in the "empty dot" side of the (0,2) configuration. If it had been localized in the blocked dot (the "2" side of the (0,2) configuration), it would provide a low-energy triplet that lifts the spin blockade.

The calculated eigenvalues of the matrix at point R (i.e. in the PSB regime, with small positive detuning ε) are given in Supplementary Fig. 2b, showing the seven eigenstates as a function of the magnetic field B. The spectral curves arising from the transitions between any two states can be mapped to this energy spectrum. Energetically, spin blockade happens when the low-energy (11) triplet $|\downarrow\downarrow\rangle$ is occupied, and spin-flip transition from this state to any other would lead to a lift of PSB and an increase in current, and thus an EDSR signal. The high-energy triplet $|\uparrow\uparrow\rangle$ is usually unoccupied, especially at higher magnetic field, thus transitions originating from it are generally not observed experimentally. On the other hand, at a very low field it does mix with S_{02} significantly by spin-flip tunneling, such that a second-order two-spin-flip transition can be seen in the experimental measurement, though that particular signal quickly fades away as the magnetic field is increased. Moreover, two excited-state related resonances are included in the spectrum as well (blue and red curves in Supplementary Fig. 2a).

When we measure Rabi oscillations and Ramsey fringes of the spins, the working position is fixed at a large value of detuning (point M in Fig. 1b of the main text) deep in the Coulomb blockade

regime, instead of point R in the low-detuning regime (ε~0) where the EDSR spectrum Fig.1d (main text) is measured. To clarify the manipulated spin states more clearly, we also perform the spectral calculation in Supplementary Fig. 2 c & d by assuming $\varepsilon \sim -100$ μeV, where the (1,1) states are well decoupled from the S_{02} singlet.

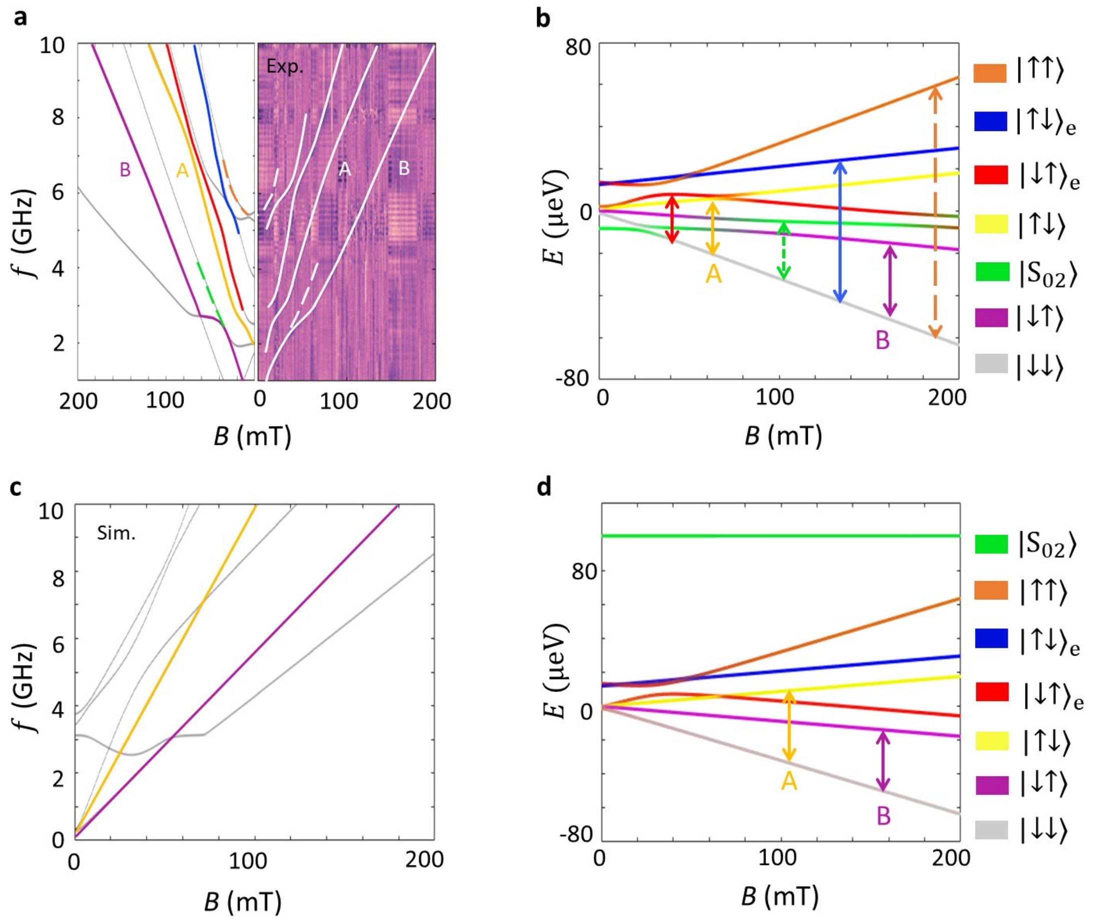

Supplementary Figure 2: EDSR spectrum. a. Simulated and experimental results of the EDSR spectrum. Left panel: calculated resonances between |↓↓⟩ and other six states (two dashed and four solid curves). These colored resonance are indicated by arrows in **b**. Eigenstates, as well as mixing, of these seven states vary as a function of magnetic field. Parameters used for simulation are $g_{\mathrm{L}} = 7$, $g_{\mathrm{R}} = 3.95$, $2t = 8$ μeV, $2t_{\mathrm{e}} = 8$ μeV, $\varepsilon = 5$ μeV, $|\Delta_{\mathrm{SO}}| = 1$ μeV, $\Delta_{\mathrm{e}} = 12$ μeV, $|\Delta_{\mathrm{SO}}^{\mathrm{e1}}| = 3$ μeV and $|\Delta_{\mathrm{SO}}^{\mathrm{e2}}| = 0.5$ μeV. **c & d**. Simulated spectrum and eigenstates at a large detuning $\varepsilon = -100$ μeV (Coulomb blockade regime). The coherent control of the left (right) spin is performed at the yellow (purple) resonance.

Compared to EDSR arising from intrinsic SOC, g-tensor modulation is often also considered when studying EDSR[4,5]. If we consider this effect, the effective Zeeman Hamiltonian reads

$$H_Z = \frac{1}{2}\mu\boldsymbol{\sigma}^T \cdot \hat{g} \cdot \boldsymbol{B},$$

where $\sigma = (\sigma_x, \sigma_y, \sigma_z)$ are the Pauli matrices. H_z is fully parametrized by the nine independent elements of the matrix $\hat{g}$. For a given $\boldsymbol{B} = (0,0,B_z)$, the two hole-Hamiltonian in the five ground basis evolves into

$$H_{DQD} = \begin{pmatrix} -\varepsilon & t/\sqrt{2} & -t/\sqrt{2} & -\Delta_{SO}^* & \Delta_{SO} \\ t/\sqrt{2} & (g_1^{33} - g_2^{33})\mu_B B/2 & 0 & -(g_1^{13} + ig_1^{23})\mu_B B/2 & -(g_2^{13} - ig_2^{23})\mu_B B/2 \\ -t/\sqrt{2} & 0 & -(g_1^{33} - g_2^{33})\mu_B B/2 & -(g_2^{13} + ig_2^{23})\mu_B B/2 & -(g_1^{13} - ig_1^{23})\mu_B B/2 \\ -\Delta_{SO} & -(g_1^{13} - ig_1^{23})\mu_B B/2 & -(g_2^{13} - ig_2^{23})\mu_B B/2 & -(g_1^{33} + g_2^{33})\mu_B B/2 & 0 \\ \Delta_{SO}^* & -(g_2^{13} + ig_2^{23})\mu_B B/2 & -(g_1^{13} + ig_1^{23})\mu_B B/2 & 0 & (g_1^{33} + g_2^{33})\mu_B B/2 \end{pmatrix}.$$

While the shear terms of the g-tensor could indeed be present in our system, they are ignored here considering the high symmetry growth direction for the hut wire[6], which makes our QD in the hut wire more similar to those made from two-dimensional hole gas. We thus stick to a model of SOI driven EDSR and neglect the effects of g-factor modulation in our simulations for simplicity and clarity.

The condition of resonances depends on control parameters such as the inter-dot tunnel coupling or the electrostatic field, which can be seen in Supplementary Fig. 3, where the oblique resonance varies as a function of the middle gate voltage. To verify that the Rabi frequency can be further increased, we have compared the Rabi frequency as a function of pulse height in Supplementary Fig. 4, where a deeper working position leads to a smaller Rabi frequency. Here, we obtain the two g-factors as $g_{\mathrm{L}} = 7$ and $g_{\mathrm{R}} = 3.95$. For heavy holes in Ge hut wire, one expects a small in-plane g-factor of 0.2 and a large out-of-plane g-factor of 21.4[7]. In our model, we assume two different g-factors for the spins in the left dot and the right dot respectively. We believe this difference can be attributed to the unequal hole occupations between the two dots. Our observations seem to indicate that fewer holes occupy the left dot compared to the right dot (Supplementary Fig. 1b & Supplementary Fig. 8b). It is thus quite probable that the wave function of the manipulated spin differs in the two dots. Moreover, a recent preprint[8] shows a large out-of-plane g-factor of 15.7 and very different g-factors due to a different hole filling. In short, the g-

factor difference between mode A and mode B is quite understandable considering their different occupations and states involved.

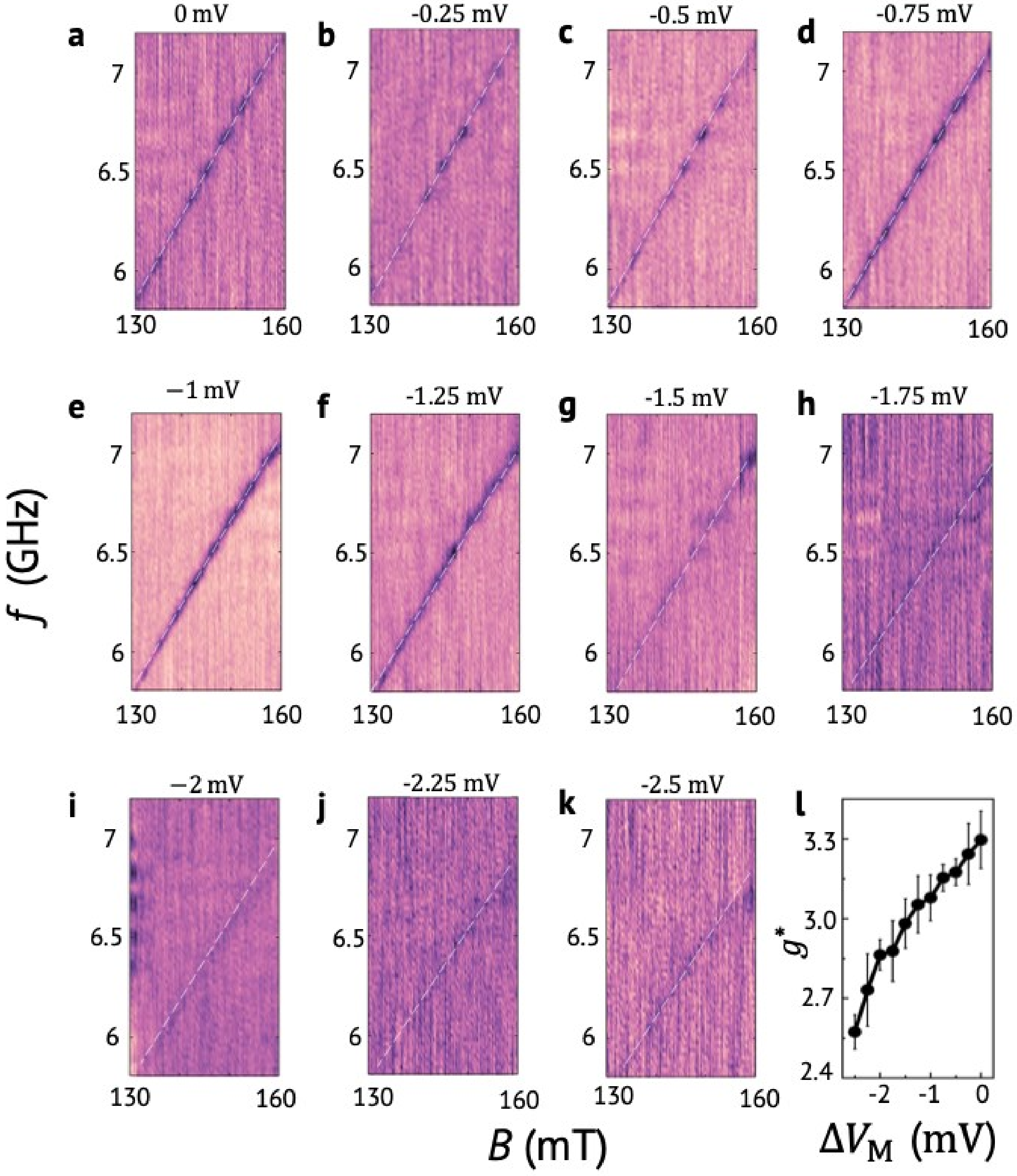

Supplementary Figure 3: EDSR of mode B at different gate voltages of gate M. The obtained $g*$-factor ($\Delta E = g^{*}\mu_{B}B$) in the linear regime varies as a function of the gate voltage of middle gate M. **a-k** correspond to the set of EDSR resonances obtained at V_{M}ranging from 0 mV to -2.5 mV. **l** shows the obtained $g*$-factor in each EDSR pattern. Error bars are from the collimation error when extracting the slopes from these resonances.

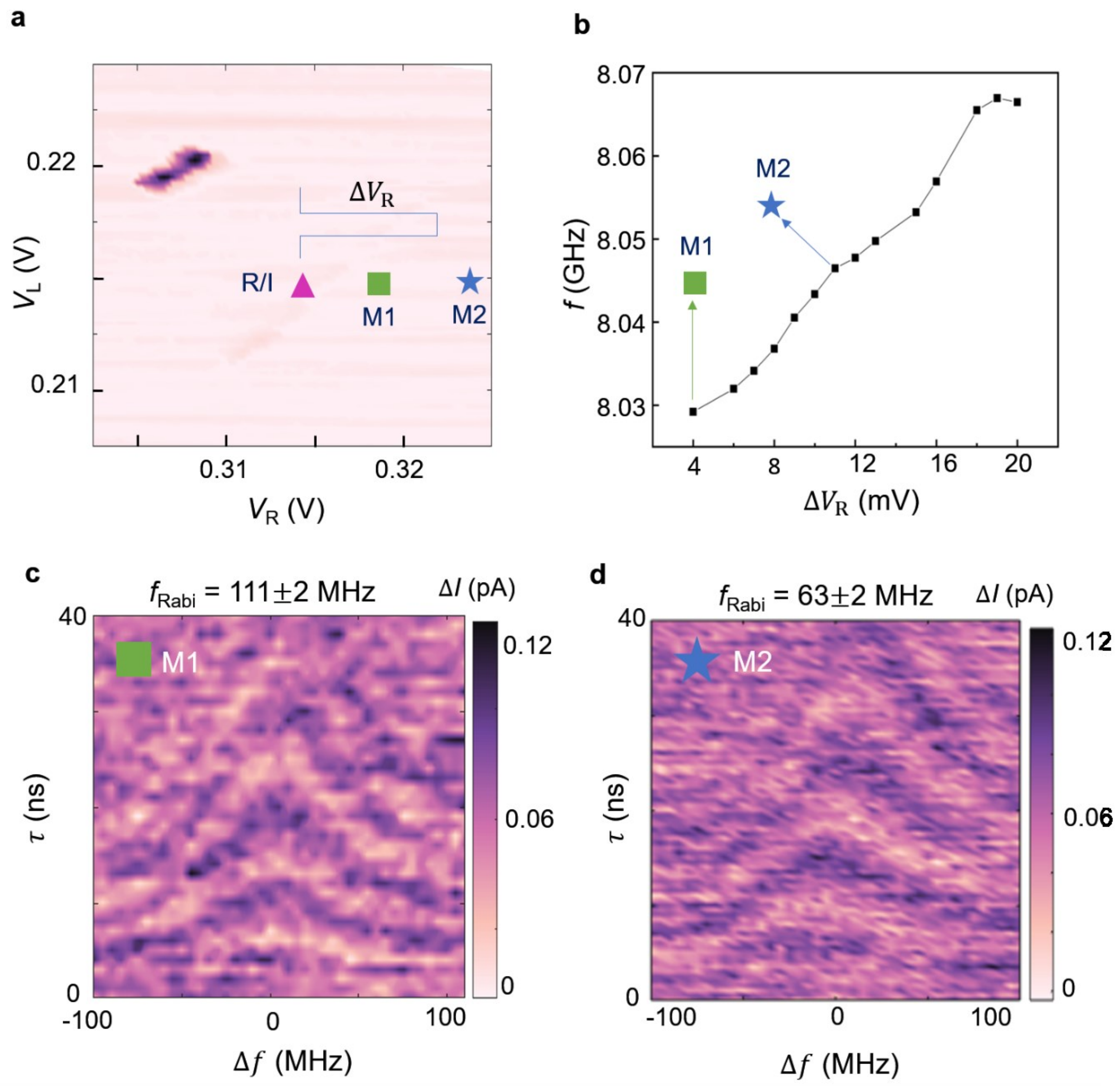

Supplementary Figure 4: Tunable Rabi frequency as a function of pulse height a. Schematic of the manipulation point in the stability diagram. **b**. Qubit frequency shifts as a function of pulse height. Each point is obtained by the chevron pattern of Rabi oscillations. The cases of M1 and M2 are shown in **c** and **d** with a different Rabi frequency. All the measurements in the main text are performed at the position of M2.

Supplementary Note 3: Noise power spectral density

To extract the noise type (α) for fitting the data for dynamical decoupling, we studied the power spectral density of noise in our system. As shown in Supplementary Fig. 5a, by repeating the Ramsey oscillation at a fixed waiting time, we obtained the time evolution of the Ramsey signal.

After extracting the frequency detuning (Δf), the power spectral density as a function of frequency f is mapped according to Welch's method[9]. As shown in Supplementary Fig. 5b, the noise in our system shows a $1/f^{0.9}$ dependence. We thus use $\alpha = 0.9$ to fit our data for the Hahn echo experiment.

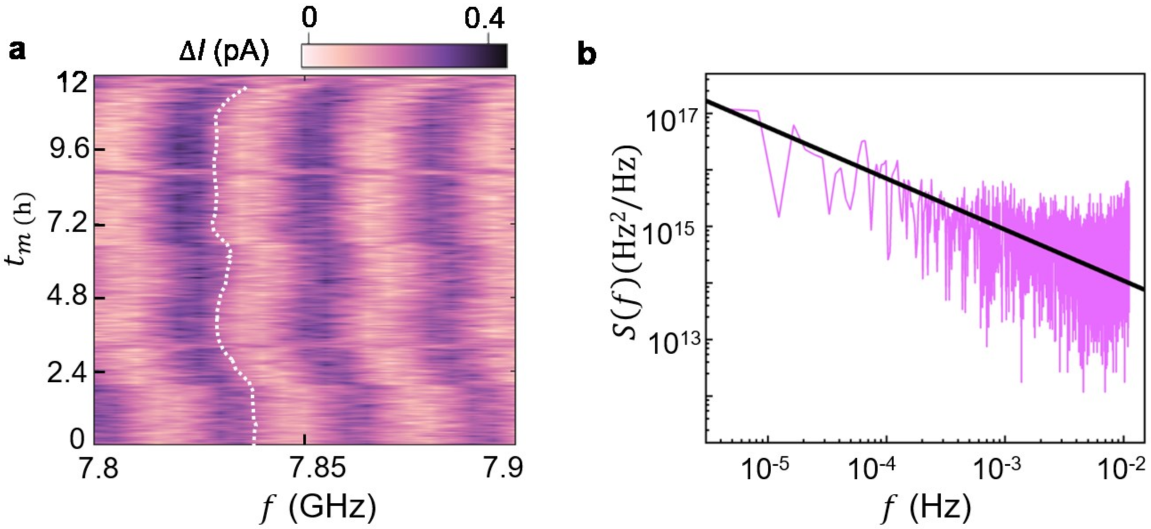

Supplementary Figure 5: Noise power spectral density. **a**, Measured spin-up probability as a function of microwave frequency f during a measuring time $t_m = 12$ h (corresponding to time evolution of the Ramsey fringe pattern at the waiting time of $\tau_{\text{burst}} = 30$ ns in Fig 3a of the main text). The white dash ($v(t)$) tracks the resonance detuning Δf extracted from the fringes. **b**, Noise spectral content $S(f)$ calculated from the detuning frequency shift $v(t)$ (dash in a). It shows a $1/f^{0.9}$ dependence below 0.01 Hz (black trace). In the calculation, we use the formula $S(f_k) = \frac{t_d}{N}\left|\sum_{n=1}^{N} v(t_n)e^{-2\pi i f_k t_n}\right|^2$ where t_d is the sampling lag, and N is the number of data points.

Supplementary Note 4: Additional information on the Rabi oscillation of mode A and dephasing time

In Supplementary Fig. 6a, the Rabi oscillations of mode A at power of -15 dBm < P < 0 dBm are measured for comparison with the result of mode B. More than 30 periods can be achieved even when $\tau_{\text{burst}}$is longer than 200 ns. The experimental results are transformed into the frequency domain to identify the dependence of the Rabi frequency on the power (shown in Fig. 4a of the main text). Rabi oscillations at $P = 10$, 11 and 12 dBm are shown in Supplementary Fig. 6b. The

signals deteriorate dramatically as the power increases, which we attribute as consequences of PAT and heating by the microwave. Furthermore, as shown in Supplementary Fig. 6c, the Rabi frequencies extracted from these three oscillations do not fit the linear dependence anymore and we attribute this to the PAT effect as discussed in the main text.

To exclude the effect of different power while comparing the results of mode A and mode B, we measured the dephasing time of the two modes at the same power of P = 0 dBm respectively. As shown in Supplementary Fig. 6d, $T_2^* = 66 \pm 6\ ns$ is obtained for mode A and is in accord with the result in Fig. 3e of the main text (result of mode B). We can then conclude that dephasing does not change in these two different modes. In the case of a very strong microwave burst when P is above 9dBm in the same mode, the fast decay of Rabi oscillations (Supplementary Fig. 6b) might be explained by PAT. A long microwave burst of strong microwave field helps the tunneling of a hole in either of the dots to reservoirs or between the dots by absorbing photons, which could result in the lift of spin blockade. This process would lead to an increase of leakage to non-qubit states and accelerated decay of Rabi oscillations, which mainly affects spin manipulation. When the driving power P is increased to 10, 11 and 12 dBm, Rabi oscillation decays too fast for us to consistently generate the $\pi/2$ pulse with reasonable fidelity. We are thus unable to obtain T_2^* in these high-power cases. However, we did obtain T_2^* at lower powers (Supplementary Fig. 6d) where T_2^* shows a downward trend as driving power increases, when -5 dBm < P < 9dBm. Based on these results, we believe that the value of T_2* at P=10, 11 and 12 dBm would be further reduced.

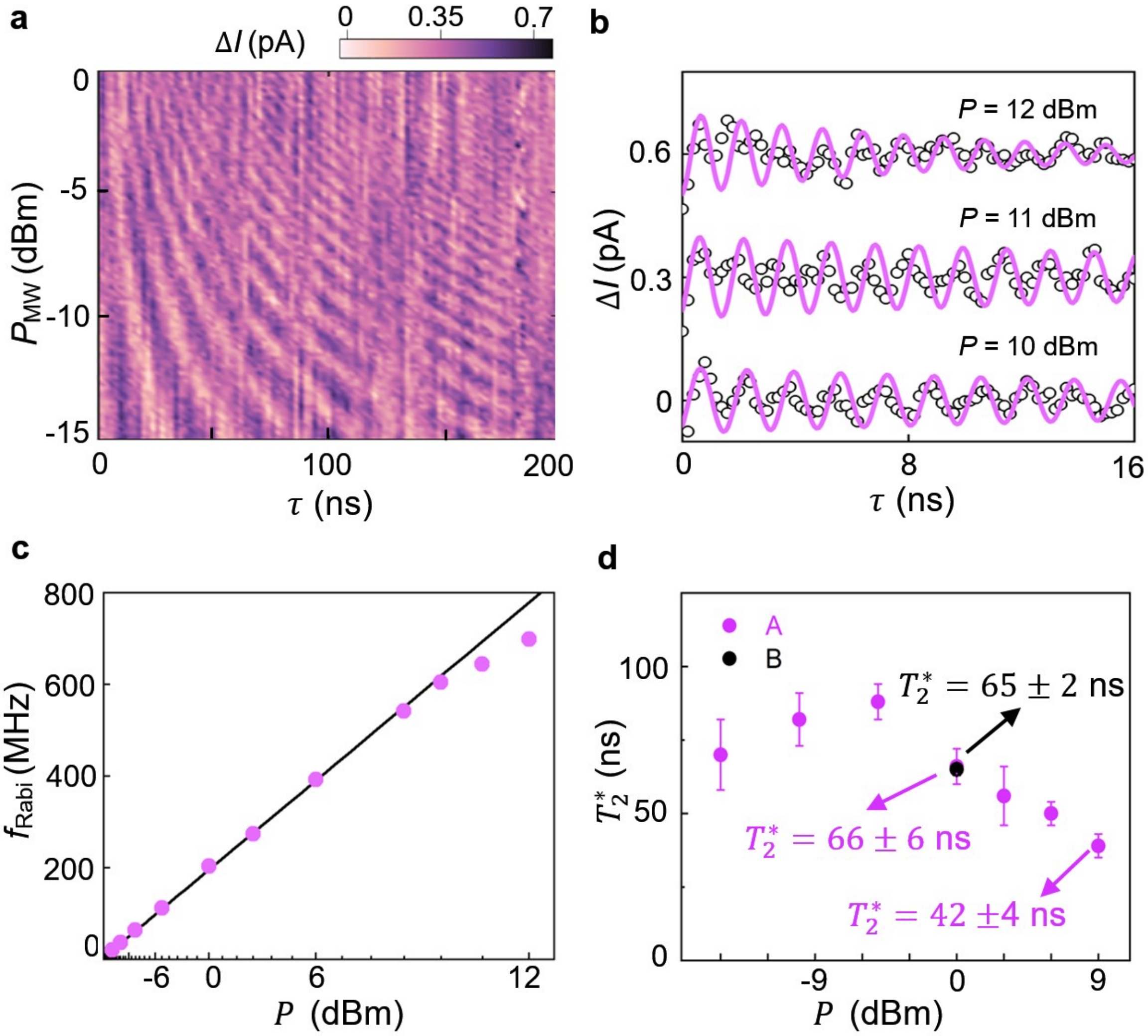

Supplementary Figure 6: Additional information of mode A. a, Rabi oscillations of the qubit of mode A at low microwave power of $-15 < P < 0$ dBm. **b**, Rabi Oscillations at higher microwave power. Rabi frequencies are obtained at 604 ± 2, 644 ± 2, 698 ± 2 MHz from bottom to top. **c**, When microwave power $P > 9$ dBm, the amplitude damps and the extracted frequencies do not fit the linear dependence on $\sqrt{P}$ anymore. **d**, Dephasing time of mode A at different microwave power. $T_2^* = 66 \pm 6$ ns and $T_2^* = 42 \pm 4$ ns are extracted from Ramsey experiment at $P = 0$ dBm and $P = 9$ dBm respectively $T_2^* = 65 \pm 2$ ns of mode B is measured at $P = 0$ dBm with error bars from Gaussian fits.

Supplementary Note 5: Two-axis control of mode B

5.1 Rabi oscillations

We performed two-axis control of mode B as well as mode A. To characterize the x-rotation, a chevron pattern (Supplementary Fig. 7.1a) in the Rabi experiment is obtained at the power $P = -15$ dBm. Supplementary Fig. 7.1b shows Rabi oscillations at power $-15 < P < 0$ dBm. Similar to Supplementary Fig. 6a, these oscillations are Fourier transformed to obtain the Rabi frequency as shown in Fig. 4a (main text). The data for the Rabi frequency results of mode B at $P > 0$dBm in Fig. 4a (main text) is extracted from Supplementary Fig. 7.1c. Even at the power of 9 dBm, clear periodic oscillations are still observable.

5.2 Spin relaxation time

We characterized hole spin relaxation by realizing spin-to-charge conversion in a cyclic sequence switching between Coulomb blockade and spin blockade (Supplementary Fig. 7.1d, inset). Two thirds of the cycle is spent in the Coulomb blockade regime for readout and initialization. The remaining part is used for pumping the spin to the excited state with a π pulse. At the magnetic field of $B = 156$ mT, we obtained a hole spin relaxation time of 3.65 μs in Supplementary Fig. 7.1d. This is the first time hole spin relaxation is characterized at a magnetic field lower than 200 mT. Additionally, we believe that spin relaxation in our sample is long enough such that the amplitude decay of Rabi and Ramsey oscillations are not dominated by this process.

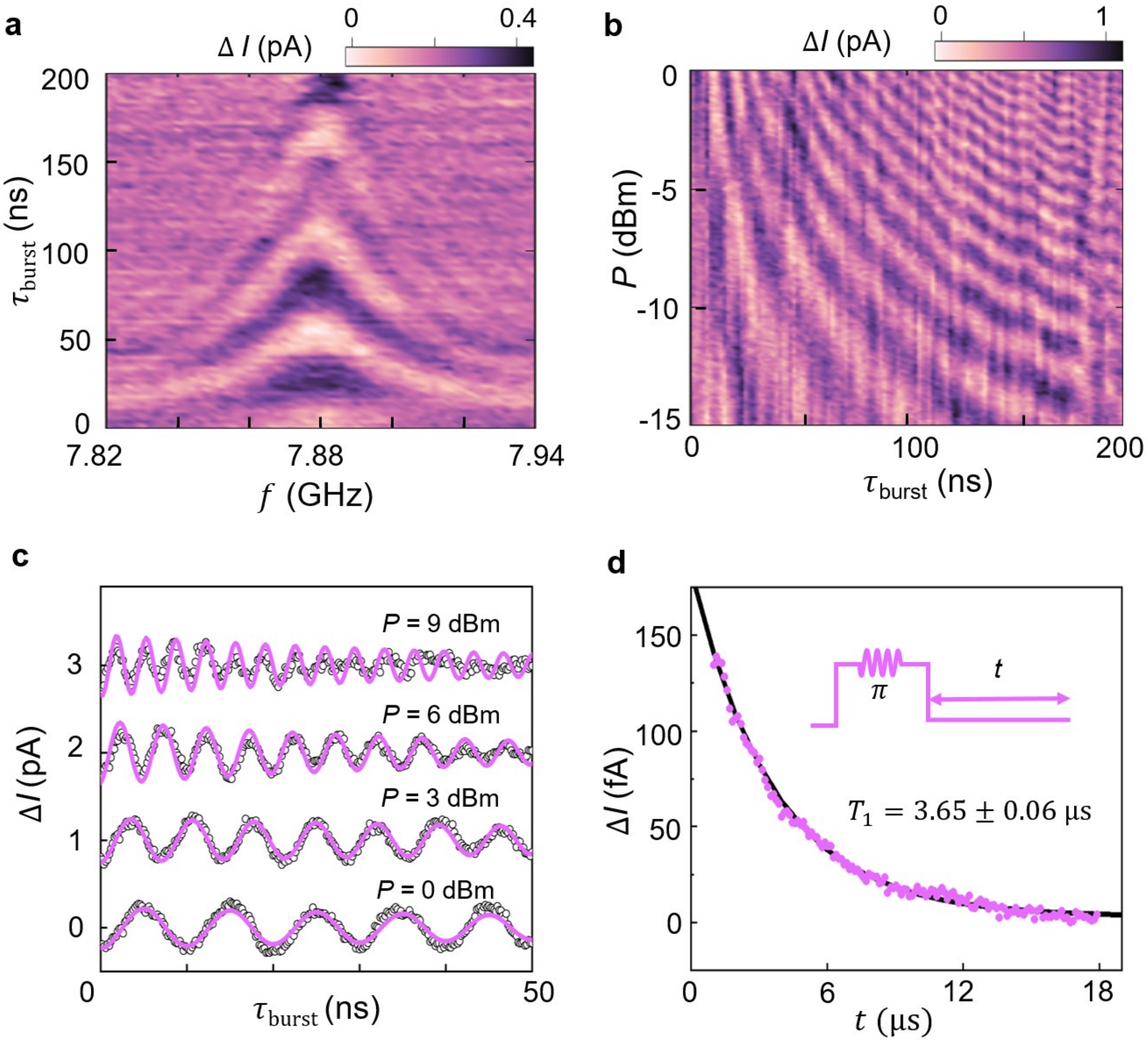

Supplementary Figure 7.1: Rabi oscillations of mode B and extracted relaxation time. a, Chevron pattern under the driving power of $P = -15$ dBm at B = 156 mT. **b**, Oscillations of the qubit of mode B at f = 7.88 GHz under low microwave power of $-15 < P < 0$ dBm. **c**, Rabi oscillations at $f = 7.88$ GHz with fits to $A \cdot cos(f_R \tau_{burst} + \varphi) \cdot \exp(-(\tau_{burst}/T_2^R)^2) + I_0$ (An offset of 1pA is set between two oscillations for clarity. Rabi frequencies are 100 ± 0.2, 140 ± 0.3, 202 ± 0.5 and 291 ± 0.9 MHz from bottom to top). **d**, A spin relaxation time $T_1 = 3.65 \pm 0.06$ μs is obtained by fitting the decay process using $\Delta I = I_0 + A \cdot \exp(-\frac{t}{T_1})$. The state is prepared to the excited with a π pulse during measurement.

5.3 Ramsey experiment

Ramsey fringe measurement of mode B is performed at $P = 0$ dBm. At $B = 156\text{mT}$, a Ramsey fringe pattern is depicted in Supplementary Fig. 7.2a. Similar to mode A, a Fourier transform is made to identify the frequency dependence in Supplementary Fig. 7.2b.

In Supplementary Fig. 7.2c, the average current is measured by varying the frequency of applied microwaves and the relative phase between the two $\pi/2$ pulses. Similar to Fig. 3c (main text), the relative phase of the second pulse controls the rotation axis during measurement.

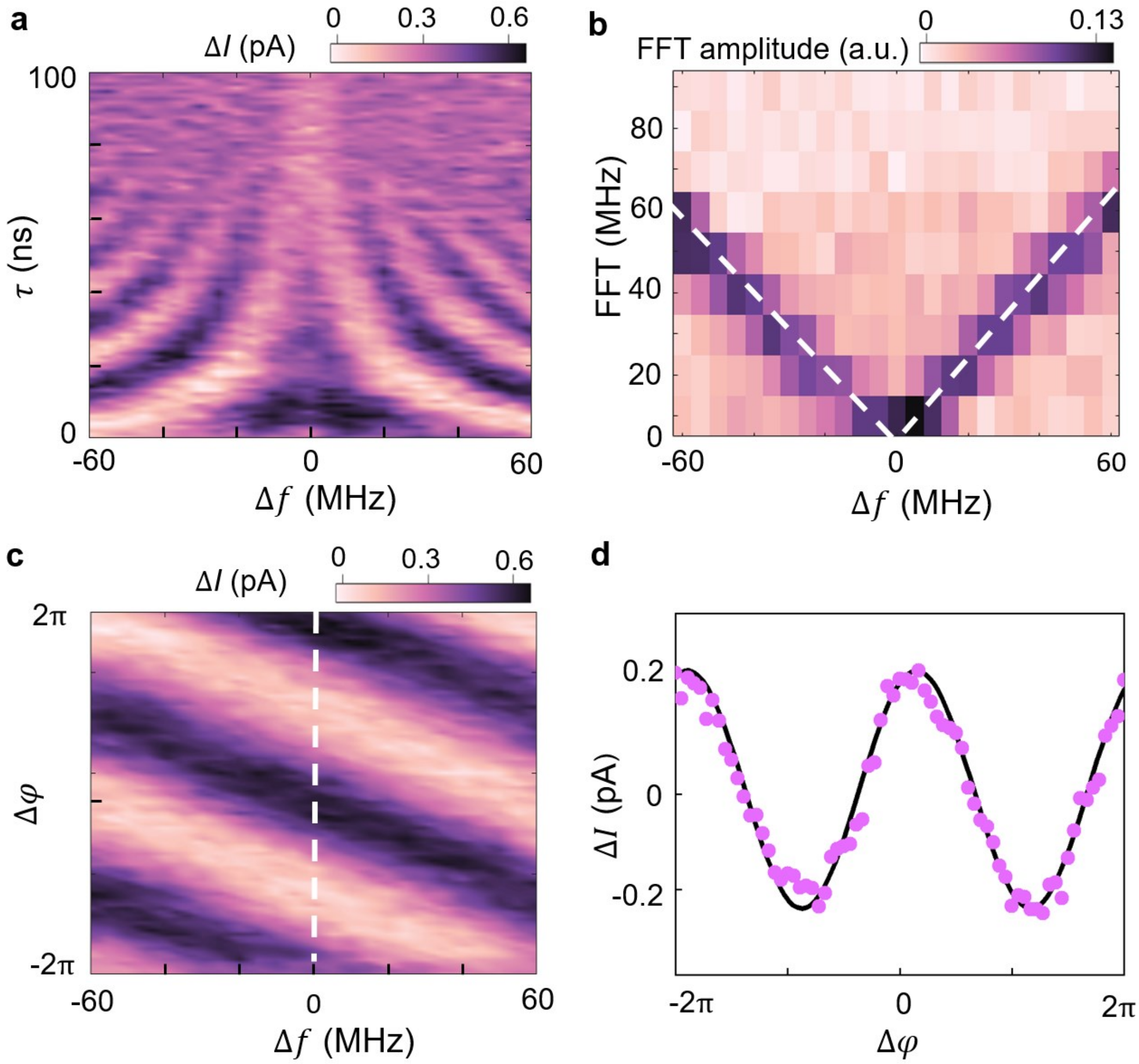

Supplementary Figure 7.2: Ramsey data of mode B. a, Transport current of mode B as functions of microwave frequency detuning $\Delta f = f - f_0$ (f is the microwave frequency and $f_0 = 7.88$ GHz is the qubit the frequency) and free evolution time τ between the two $\pi/2$ pulses for the Ramsey

fringe measurement at microwave power of $P = 0$ dBm. **b**, Frequency Fourier transform corresponding to **a**. Two white dash lines mark the dependence of Ramsey frequency f_{Ramsey} on frequency detuning Δf. **c**, Phase control of mode B. The phase is controlled by the second $\pi/2$ pulse (2.5 ns) and a period of 2π from the inset (along dash line) shows the perfect result. **d**, Oscillation along the dash in **c** with a cycle value of 2π.

Supplementary Note 6: Simulation of static electric field E_{dc} and effective driving field E_{ac}

To calculate the spin-orbit length, we have to know the true electric driving field E_{ac}. We simulate the applied a.c. field from microwave by COMSOL. The device structure is obtained from our design drawing and checked by the SEM image (Supplementary Fig. 8a). We then input the static voltages on the gates to confirm the positions of our dots. From the results shown in Supplementary Fig. 8b, we conclude that our dots are underneath gate L and gate R, where the static field is lower than the surrounding areas. The effective driving field due to the microwave are calculated similarly. Here, the a.c. fields along the *x* and *y* directions in Supplementary Fig. 8c & d accounts for the effective driving while the *z*-component of the field does not contribute to EDSR (Supplementary Fig. 8e). For example, at the power of $P = 0$ dBm, AC electric fields in the left (right) dot are $E_{ac}^{x} = 230\ \mathrm{V/m}\ (715\ \mathrm{V/m})$ and $E_{ac}^{y} = 3475\ \mathrm{V/m}\ (1810\ \mathrm{V/m})$ and $E_{ac} = \sqrt{(E_{ac}^{x})^2 + (E_{ac}^{y})^2}$ is used in the main text. Supplementary Fig. 8f shows the value of a.c. field along the nanowire (i.e. *y* direction) and its gradient, which drives holes in both dots to move back and forth mainly along the nanowire due to the larger component along y direction. Using our dot parameters, we find a field-induced displacement of 15 pm in the right dot as shown by Supplementary Fig. 8 g & h, larger than the micromagnet induced displacement of 4 pm in silicon QD[10,11].

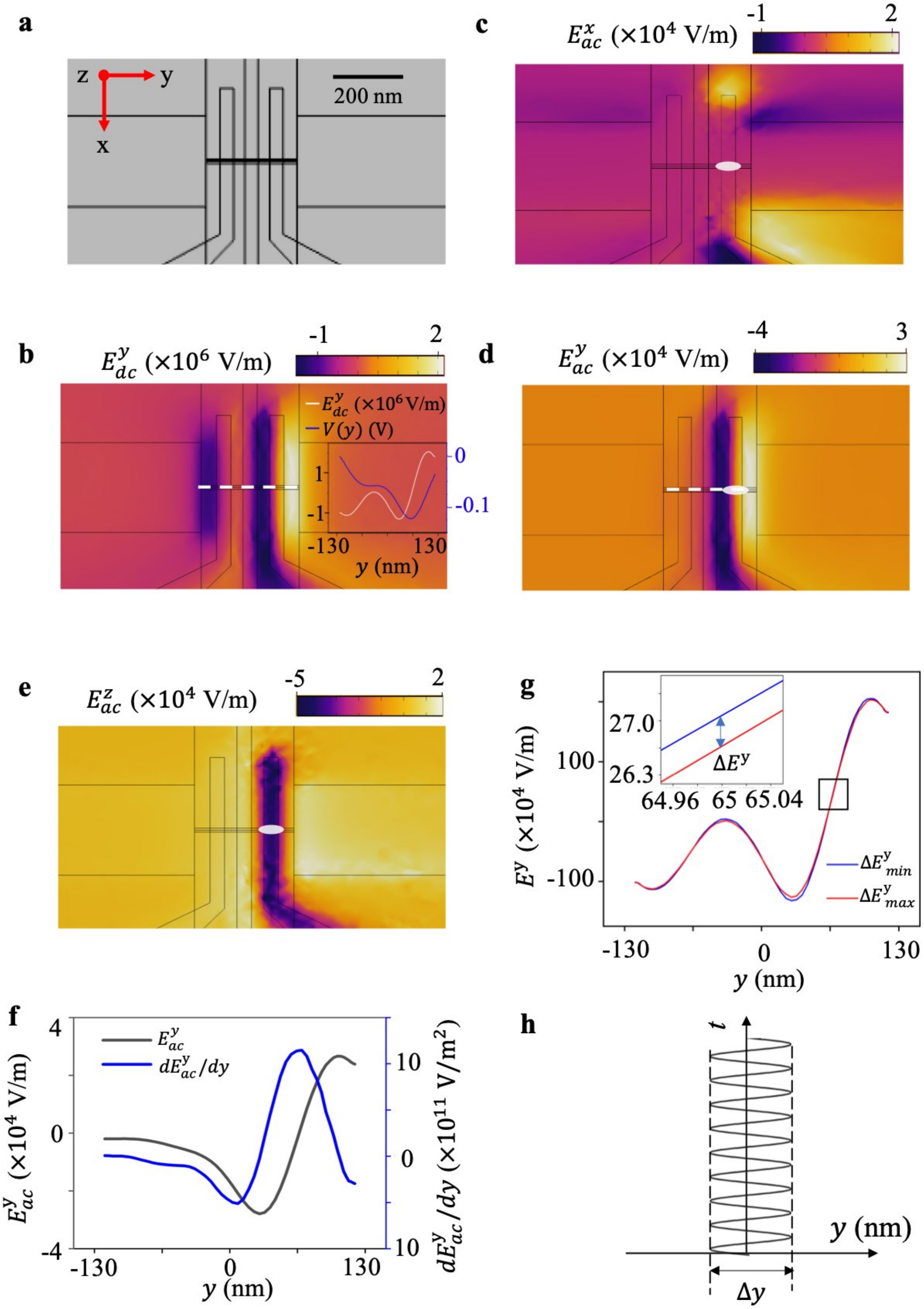

a
z
y
x
200 nm
b
E_{dc}^{y} (×10⁶ V/m)
-1
2
E_{dc}^{y} (×10⁶ V/m)
V(y) (V)
0
-0.1
1
-1
-130
130
y (nm)
c
E_{ac}^{x} (×10⁴ V/m)
-1
2
d
E_{ac}^{y} (×10⁴ V/m)
-4
3
e
E_{ac}^{z} (×10⁴ V/m)
-5
2
f
E_{ac}^{y}
dE_{ac}^{y}/dy
E_{ac}^{y} (×10⁴ V/m)
dE_{ac}^{y}/dy (×10¹¹ V/m²)
4
0
-4
10
0
10
-130
0
130
y (nm)
g
E^{y} (×10⁴ V/m)
100
-100
27.0
26.3
64.96
65
65.04
ΔE^{y}
ΔE_{min}^{y}
ΔE_{max}^{y}
-130
0
130
y (nm)
h
t
y (nm)
Δy

Supplementary Figure 8: Comsol simulation. a, Device structure from SEM image for input of size in COMSOL. **b**, Simulated y-component of static electric field E_{dc}^{y} after applying voltages of $V_{\mathrm{L}} = 0.17$ V, V_{M} =0.085 V, $V_{\mathrm{R}} = 0.355$ V and $V_{\mathrm{sd}} = 3$ mV. Inset: E_{dc}^{y} and obtained electrostatic potential distribution $V(y)$ as a function of position along y direction (linecut along the dash line). **c, d &e,** Simulation results along *x*, *y* and *z* axes after applying an alternating electric field at $P =$ 0 dBm. The white ellipse marks the position of the right dot. **f**, E_{ac}^{y} and dE_{ac}^{y}/dy as a function of position along y direction in **d. g,** The whole electric field $E^{y} = E_{\mathrm{ac}}^{y} + E_{\mathrm{dc}}^{y}$ shifts along y direction due to the microwave. We extract right-dot shift of $\Delta E^{y} \approx 3600\ \mathrm{V/m}$ at the position underneath gate R. **h,** Using this value, we can deduce the dot displacement $\Delta\mathrm{y} = \frac{eE_{ac}a_{\mathrm{x}}^{2}}{\hbar\omega_{y}}$ and the effective magnetic field $B_{ac} = 2B \cdot \frac{a_{\mathrm{x}}}{l_{\mathrm{so}}} \cdot \frac{eE_{ac}a_{\mathrm{x}}}{\hbar\omega_{y}}$ due to the driving by the a.c. electric field. We find $\Delta y =$ 15 pm (i.e. Δr in Figure 1c) and $B_{ac} = 3.6$ mT for the right dot spin at the driving power of $P =$ 0 dBm, consistent with the 100 MHz Rabi frequency for mode B at this power with a spin-orbit length of 1.4 nm.

Supplementary Note 7: Calibration of the phase of second pulse in Ramsey fringe pattern

During the Ramsey measurement, the fringe pattern is determind by the phase of the second pulse. To detect a pure decay at zero frequency detuning, the phase difference between the first and the second $\pi/2$ pulse should be controlled precisely until $\Delta\varphi = 0$ is satisfied. We achieve this by controlling a continuous waveform without phase shift with a sinusoidal waveform of IQ input from our AWG.

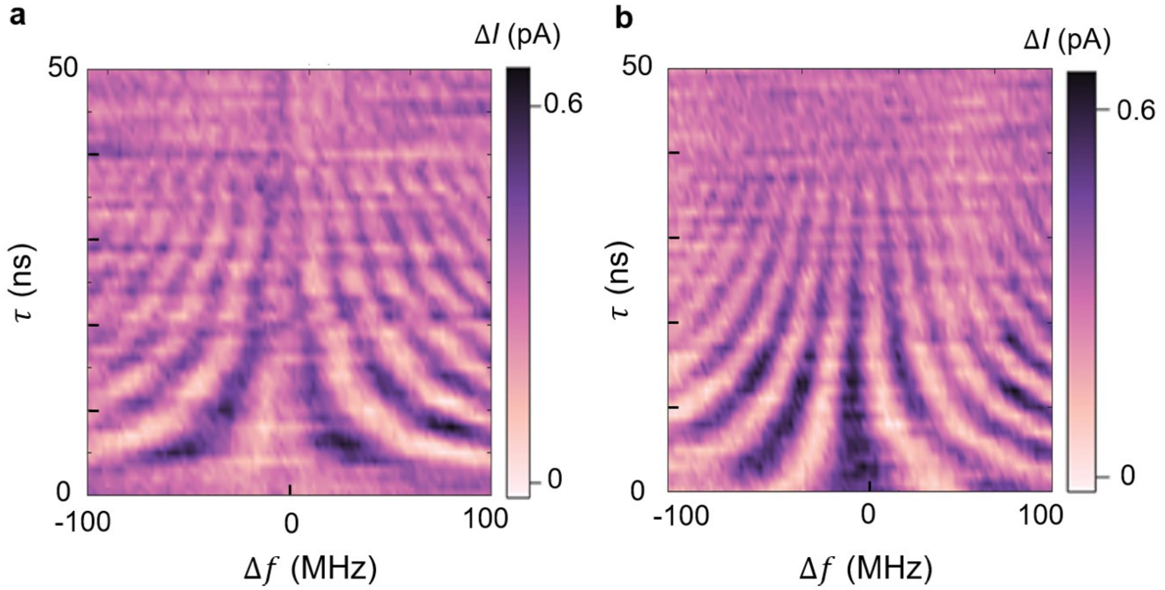

Supplementary Figure 9: Calibration of the phase of second pulse in Ramsey fringe pattern. **a**, Ramsey fringe pattern of mode B, obtained at the microwave power $P = -10$ dBm with the phase difference between the first and the second $\pi/2$ pulse $\Delta\varphi = \pi/2$. **b**, Similar Ramsey fringe pattern when $\Delta\varphi = 0$. It is clear that the spin decays from excited state to ground state at the frequency detuning $\Delta f = 0$ while it remains in ground states in **a**.

Supplementary Note 8: References on different quantum dot spin systems

There are many ways to evaluate the quality of a qubit or its manipulation. Two good ways are gate tomography and fidelity via randomized benchmarking. Tomography is a good gauge of the system and its coherence terms, and has been used in some previous works, though it takes a great deal of time to obtain the density matrix elements. Gate fidelity is comparatively easier to obtain and is measured in many experiments. Here we would like to evaluate how many operations can be performed before a qubit decoheres. Thus we focus on the x-axis rotation speed and coherence time of different spin qubits in previous literatures. Please note that we use the values of T_2^* only for qualitative comparison, because it may also depend on the microwave power which can induce noise during a measurement. In short, our simple comparison here is only meant to show the general characteristics of different spin qubits.

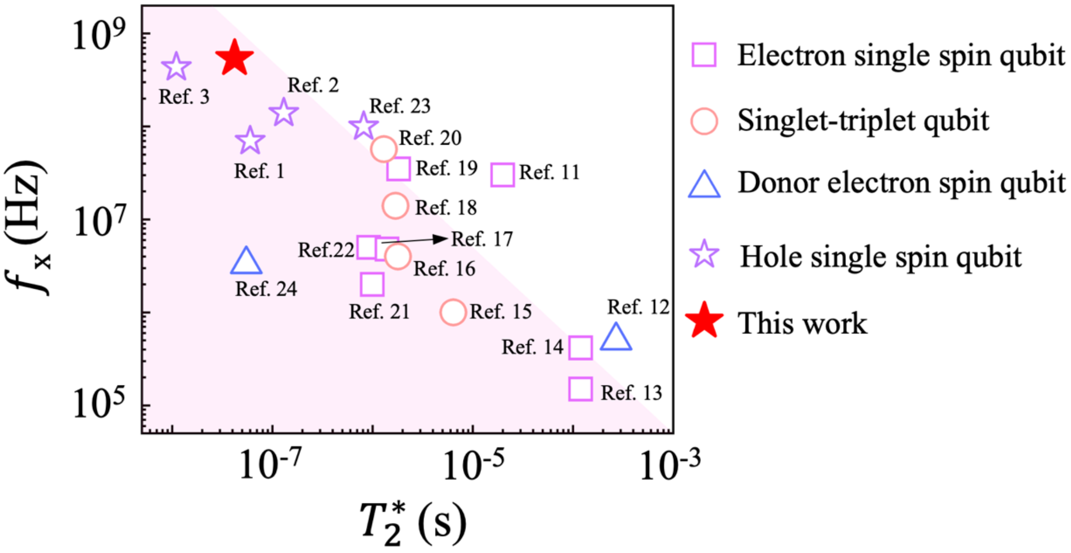

Supplementary Figure 10: Qualitative benchmarking of dephasing time and *x*-rotation speed for different types of spin qubits. Related works from the same group are not listed.

Supplementary Note 9: Heavy hole states in GHW

Considering the HH and LH bands of Ge and assuming that the HW is free of shear strain, the Hamiltonian for a two-dimensional quantum-dot-confined hole in the presence of a magnetic field is[25]

$H = \frac{\hbar}{2m}\left[\left(\gamma_1 + \frac{5\gamma_2}{2}\right)k^2 - 2\gamma_2 \sum_\nu k_\nu^2 J_\nu^2 - 4\gamma_3\left(\{k_x, k_y\}\cdot\{J_x, J_y\} + \mathrm{c.p.}\right)\right] + 2\mu_B \boldsymbol{B}\cdot(\kappa\boldsymbol{J} + q\boldsymbol{J^3}) +$
$b\sum_\nu \epsilon_{\nu\nu} J_\nu^2 + V(x, z)$.

It consists of the Luttinger-Kohn Hamiltonian, the Bir-Pikus Hamiltonian and the confinement in the transverse directions $V(x, z)$. We follow the calculation in Ref. 7 and Ref. 26, and treat our potential as a rectangle hard-wall potential of width L_x and height L_z for simplicity, i.e. $V(x, z) = 0$ if $|x| < \frac{L_x}{2}$ and $|z| < \frac{L_z}{2}$ and $V(x, z) = \infty$ otherwise[25], where the three axes x, y, z are oriented along the width, length and height, respectively, of the HW. The applied magnetic field is $\boldsymbol{B} = (B_x, B_y, B_z)$, and the kinetic momentum is $\hbar k = -i\hbar/\nabla + e\boldsymbol{A}$ where $\boldsymbol{B} = \boldsymbol{\nabla}\times\boldsymbol{A}$. The vector potential is chosen as $\boldsymbol{A} = (B_y z - B_z y, -\frac{B_x z}{2}, \frac{B_x y}{2})$ for convenience. We use the basis of

$$\left|j_z, n_x, n_y, n_z\right\rangle = |j_z\rangle \otimes |\varphi_{n_x,n_y,n_z}\rangle$$

with the envelope function

$$\varphi_{n_x,n_y,n_z}(x, y, z) = \frac{2}{\sqrt{L_x L_z}} \sin\left[n_x\pi\left(\frac{x}{L_x} + \frac{1}{2}\right)\right] \times \sin\left[n_y\pi\left(\frac{y}{L_y} + \frac{1}{2}\right)\right] \times \sin\left[n_z\pi\left(\frac{z}{L_z} + \frac{1}{2}\right)\right].$$

We then project the Hamiltonian to a subspace with $n_x \leq 3$, $n_y \leq 3$ and $n_z \leq 3$, using the band structure parameters of bulk Ge $\gamma_1 = 13.35, \gamma_2 = 4.25, \gamma_3 = 5.69, \kappa = 3.41, q = 0.07$. The values for the strain tensor elements are $\epsilon_{xx} = \epsilon_{yy} = -0.033$ and $\epsilon_{zz} = 0.02$[27].

Using a magnetic field of 100 mT (close to mode A and B) along the z direction as in the experiment, $L_x = 5$ nm, $L_y = 40$ nm from Comsol simulation (inset of Supplementary Fig. 8b), $L_z \leq 2$ nm and other parameters, we diagonalize the (108×108) matrix of the Hamiltonian, and obtain a spin expectation value of $\langle J_z \rangle \approx 1.45$, from which we find the probability of HH to be $p_{HH} \approx 95\%$from $\frac{3}{2}p_{HH} + \frac{1}{2}(1 - p_{HH}) \approx 1.45$. Clearly, this is a nearly pure heavy hole system. Moreover, a large LH and HH splitting of $\frac{2\gamma_2\hbar^2\pi^2}{mL_z^2} \geq 1.6$ eV is obtained with $L_z \leq 2$ nm according to Ref. 7, making it unlikely to have significant LH component for any low-energy hole states.

We have also considered the case when an in-plane magnetic field is applied. Similar to the case above, we find a 95% HH probability when $B = 0.1$T. Therefore, we can conclude that the holes in our system are close to pure heavy holes no matter what the applied magnetic field direction is.

Supplementary Note 10: Heavy hole spins in GHW quantum dots

In what follows we provide a brief sketch of the principal physical ingredients needed for a qualitative understanding of the physics of spin-3/2 heavy holes confined to a quantum dot in a Ge hut wire. The full description, including a proper treatment of the orbital terms induced by the magnetic field, is deferred to a forthcoming publication. The planar system, controlled by a top-gate electric field E_g^z, is described by the following effective Hamiltonian

$$H = H_0 + V(x,y) + H_{so} + H_z + H_E,$$

where the kinetic energy and confinement potential take the form

$$H_0 + V(x,y) = \frac{(-i\hbar\nabla - e\boldsymbol{A})^2}{2m} + \frac{m(\omega_x^2 x^2 + \omega_y^2 y^2)}{2},$$

with $\boldsymbol{A}$ the vector potential and m the in-plane effective mass. The confinement is asymmetric in the x and y directions with confinement energies $\hbar\omega_x$ and $\hbar\omega_y$ respectively, and radii given by $a_{x,y}^2 = \hbar/(m\omega_{x,y})$ in the absence of a magnetic field. The eigenstates of $H_0 + V(x,y)$ are the two-dimensional harmonic oscillator states, the ground state taking the form

$$\phi_0 = \frac{1}{\sqrt{\pi a_x a_y}} \exp\left(-\frac{x^2}{2{a_x}^2}\right) \exp\left(-\frac{y^2}{2{a_y}^2}\right).$$

The Zeeman interaction is $H_z = \frac{g\mu_B B}{2}\sigma_z$, and $H_E = e(E_{ac}^y y + E_{ac}^x x)\cos(\omega t)$ represents the interaction with an in-plane electric field employed for EDSR. The static magnetic field defines the spin quantization axis. Its out-of-plane orientation takes advantage of the large g-factor for the heavy holes. The leading (spherical) contribution to Rashba spin-orbit coupling in a two-dimensional Ge hole gas is given, in terms of the wave vector, by the following expression[28]

$$H_{so} = i\alpha_2(k_+^3\sigma_- - k_-^3\sigma_+)$$

where $k_+ = k_x + ik_y$, $k_- = k_x - ik_y$, $\sigma_+ = (\sigma_x + i\sigma_y)/2$ and $\sigma_- = (\sigma_x - i\sigma_y)/2$. The parameter α_2 is determined by a combination of the Luttinger parameters, top gate electric field E_g^z, and heavy hole–light hole splitting. An additional term α_3 arising from the cubic symmetry of the Ge lattice is important in a circular quantum dot[29], but should play only a minor role in the hut wires discussed here, and will not be considered in detail.

We wish to identify an approximate spin-orbit length, which helps to parametrize the EDSR strength in our HW QD. To this end, considering the wire transport direction parallel to y axis,

we may replace k_x^2 in H_{so} by its average $\langle k_x^2 \rangle \propto 1/(a_x^2)$. Next, we retain only the leading order term in k_y, which we can write as $H_{so}^{1D} \approx \alpha_{eff}\sigma_x k_y$, with $\alpha_{eff} \approx \frac{6\alpha_2}{a_x^2}$. In this way, in analogy with the customary Rashba Hamiltonian for a two-dimensional electron gas, we define an approximate spin-orbit length $l_{so} \approx \frac{\hbar^2}{m\alpha_{eff}}$. We then expect the Rabi frequency $f_{\mathrm{Rabi}} \propto 1/l_{so}$. Note that fast control of hole spin arising from strong spin-orbit coupling which is not only related to the small spin-orbit length but also determined by other effects, such as transverse size or excited states with small energy splitting. However, as we discussed in Supplementary Note 2, we believe the low-energy excited states that are present in our double dot is localized in one of the two dots, such that it would only enhance the EDSR Rabi frequency in that dot. The fact that Rabi frequencies in both mode A and mode B are high (540 MHz and 290 MHz at 9dB driving power) is thus a likely consequence that our system has strong spin-orbit coupling strength (thus short l_{so}).

Supplementary References:

1. Maurand, R. et al. A CMOS silicon spin qubit. *Nat. Commun.* **7**, 13575 (2016).
2. Watzinger, H. et al. A germanium hole spin qubit. *Nat. Commun.* **9**, 3902 (2018).
3. Froning, F. N. M. et al. Ultrafast hole spin qubit with gate-tunable spin-orbit switch functionality. *Nat. Nanotechnol.* **16**, 308-312 (2021).
4. Tanttu, T. et al. Controlling spin-orbit interactions in silicon quantum dots using magnetic field direction. *Phys. Rev. X* **9**, 021028 (2019).
5. Voisin, B. et al. Electrical control of g-factor in a few-hole silicon nanowire MOSFET. *Nano lett.* **16,** 88-92 (2016).
6. Zhang, T. et al. Anisotropic g-Factor and Spin–Orbit Field in a Germanium Hut Wire Double Quantum Dot. *Nano Lett.* **21**, 3835−3842 (2021).
7. Watzinger, H. et al. Heavy-hole states in germanium hut wires. *Nano lett.* **16**, 6879-6885 (2016).
8. Miller, Andrew J. et al. Effective out-of-plane g-factor in strained-Ge/SiGe quantum dots. *arXiv*: 2102.01758 [cond-mat.mes-hall].
9. Welch, P. The use of fast Fourier transform for the estimation of power spectra: a method based on time averaging over short, modified periodograms. *IEEE Transactions on audio and electroacoustics* **15**, 70-73 (1967).

10. Kawakami, E. et al. Gate fidelity and coherence of an electron spin in an Si/SiGe quantum dot with micromagnet. *PNAS* **113**, 11738-11743 (2016).
11. Yoneda, J. et al. A quantum-dot spin qubit with coherence limited by charge noise and fidelity higher than 99.9% *Nat. Nanotechnol.* **13**, 102-106 (2018).
12. Muhonen, J. T. et al. Storing quantum information for 30 seconds in a nanoelectronic device. *Nat. Nanotechnol*. **9**, 986–991 (2014).
13. Veldhorst, M. et al. An addressable quantum dot qubit with fault-tolerant control-fidelity. *Nat. Nanotechnol.* **9**, 981-985 (2014).
14. Veldhorst, M. et al. A two-qubit logic gate in silicon. *Nature* **526**, 410-414 (2015).
15. Eng, K. et al. Isotopically enhanced triple-quantum-dot qubit. *Sci. Adv.* **1**, e1500214 (2015).
16. Takeda, K. et al. Resonantly Driven Singlet-Triplet Spin Qubit in Silicon. *Phys. Rev. Lett.* **124**, 117701 (2020).
17. Zajac, D. M. et al. Resonantly driven CNOT gate for electron spins. *Science* **359**, 439-442 (2018).
18. Wu, X. et al. Two-axis control of a singlet-triplet qubit with an integrated micromagnet. *PNAS* **111**, 11938-11942 (2014).
19. Takeda, K. et al. A fault-tolerant addressable spin qubit in a natural silicon quantum dot. *Sci. Adv.* **2,** e1600694 (2016).
20. Harvey-Collard, P. et al. Coherent coupling between a quantum dot and a donor in silicon. *Nat. Commun.* **8**, 1-6 (2017).
21. Watson, T. F. et al. A programmable two-qubit quantum processor in silicon. *Nature* **555**, 633-637 (2018).
22. Kawakami, E. et al. Electrical control of a long-lived spin qubit in a Si/SiGe quantum dot. *Nat. Nanotechnol.* **9**, 666-670 (2014).
23. Hendrickx, N. W. et al. Fast two-qubit logic with holes in germanium. *Nature* **577**, 487-491 (2020).
24. Pla, J. J. et al. A single-atom electron spin qubit in silicon. *Nature* **489**, 541-545 (2012).
25. Csontos, D. et al. Spin-3 2 physics of semiconductor hole nanowires: Valence-band mixing and tunable interplay between bulk-material and orbital bound-state spin splittings. *Phys. Rev. B* **79**, 155323 (2009).

26. Katsaros, G. et al. Zero field splitting of heavy-hole states in quantum dots. *Nano Lett.* **20**, 5201-5206 (2020).
27. Gao, F. et al. Site-controlled uniform Ge/Si Hut wires with electrically tunable spin–orbit coupling. *Adv. Mater.* **32**, 1906523 (2020).
28. Marcellina, E., Hamilton, A. R., Winkler, R. & Culcer, D. Spin-orbit interactions in inversion-asymmetric two-dimensional hole systems: A variational analysis. *Phys. Rev. B* **95**, 075305 (2017).
29. Wang, Z. N. et al. Suppressing charge-noise sensitivity in high-speed Ge hole spin-orbitqubits. *arXiv*: 1911.11143 [cond-mat.mes-hall].
